# Folate deficiency aggravates SARS‐CoV‐2‐induced endothelial barrier disruption in *Mthfr* heterozygous knockout mice

**DOI:** 10.1002/alz.092234

**Published:** 2025-01-03

**Authors:** Meenakshi Umar, Saifudeen Ismael, Milla Volic, Gregory W Hall, Gregory J Bix

**Affiliations:** ^1^ Department of Neurosurgery, Clinical Neuroscience Research Center, Tulane University School of Medicine, New Orleans, LA USA; ^2^ Tulane Brain Institute, Tulane University, New Orleans, LA USA; ^3^ Department of Neurology, Tulane University School of Medicine, New Orleans, LA USA

## Abstract

**Background:**

Increasing evidence suggests that SARS‐CoV‐2 infection may lead to early onset and aggravation of pre‐existing vascular dementia and Alzheimer’s disease. Methylene tetrahydrofolate reductase (Mthfr) is a critical enzyme in folate metabolism, also required for optimal brain function. *Mthfr* deficient mice display cognitive impairments and neurovascular deficits and polymorphisms in *MTHFR* increases dementia risk. Intriguingly, a globally prevalent polymorphism in *MTHFR* (C677T) is linked with the COVID‐19 incidence and mortality. Many studies have reported association *of MTHFR* polymorphisms with COVID‐19 severity suggesting it as a genetic risk factor of COVID‐19, however, there is a need of mechanistic evaluation of the underlying mechanism. In this study, we investigated the role of folate deficiency due to *MTHFR* polymorphism on SARS‐CoV‐2 mediated neuroinflammation and blood‐brain barrier disruption.

**Method:**

C57 wild‐type (WT) and *Mthfr* heterozygous knockout (*Mthfr^±^*) mice were inoculated intranasally with mock or 1 × 10^4^ PFU of the mouse adapted (MA10) strain of SARS‐CoV‐2 that is capable of infecting WT mice. After 3 days post‐infection, lung and brain were collected for pathological evaluation. Additionally, mouse brain microvascular endothelial cells (bEnd.3) cultured in folate‐sufficient and folate‐deficient medium were treated with SARS‐CoV‐2 spike proteins for 24h.

**Result:**

*Mthfr^±^* mice showed slightly higher disease associated weight loss (10.5%) compared to WT mice (8.5%). In addition, lung viral load estimation indicated an elevated trend in *Mthfr^±^* infected mice (6.3±0.5 × 10^7^ copies/100ng RNA) compared to WT (5.8±0.9 × 10^7^ copies/100ng RNA). Blood‐brain barrier integrity, as measured by ZO‐1 mRNA level, was significantly reduced by MA10 infection in *Mthfr^±^* mice compared to WT and *Mthfr^±^* mice. Immunostaining further confirmed significant reduction in ZO‐1 expression in brain cortical regions of MA10 infected *Mthfr^±^* mice compared to WT infected mice. In addition, *Mthfr^+/^* mice displayed less activated microglia in comparison to WT mice. Our preliminary in vitro study showed that folate deficiency decreased Claudin‐5 and Occludin mRNA expression in bEnd.3 cells. Additionally, exposure to SARS‐CoV‐2 spike proteins showed a further decrease in Claudin‐5 expression in folate‐deficient bEnd.3 cells.

**Conclusion:**

SARS‐Cov‐2 infection may aggravate blood‐barrier disruption in the context of *Mthfr* disruption potentially increasing susceptibility to COVID‐19 morbidity and worsened post‐COVID neurocognitive effects.

